# New Tb^3+^–simvastatin optical biosensor for sensitive determination of folic acid, progesterone, testosterone and vitamin D_3_ in biological fluids

**DOI:** 10.1039/d1ra05368j

**Published:** 2021-10-06

**Authors:** Mohamed S. Attia, Amal M. Ahmed, Tarek A. Amin, Ahmed. O. Youssef, Mohammed A. Amin, Ekram H. Mohamed, Safwat A. Mahmoud, Mona N. Abou-Omar

**Affiliations:** Chemistry Department, Faculty of Science, Ain Shams University Cairo 11566 Egypt Mohd_mostafa@sci.asu.edu.eg Mohamed_sam@yahoo.com +202 1229867311 +202 1060819022; Department of Chemistry, Collage of Science, Taif University P. O. BOX 11099 Taif 21944 Saudi Arabia; Pharmaceutical Analytical, Chemistry Department, Faculty of Pharmacy, The British University in Egypt 11837 El Sherouk City Egypt; Physics Department, Faculty of Science, Northern Border University Arar Saudi Arabia samahmoud2002@yahoo.com; Department of Chemistry, Faculty of Women for Arts, Science and Education, Ain Shams University Cairo Egypt

## Abstract

An innovative, simple and cost effective Tb^3+^–simvastatin photo probe was designed and used as a core for a spectrofluorometric approach to sensitively determine four vital biological compounds in different matrices. A Tb^3+^–simvastatin complex displays a characteristic electrical band with *λ*_em_ at 545 nm with significant luminescence intensity, which is quenched in the presence of folic acid, progesterone, testosterone and vitamin D_3_ at four variant sets of pH: 5.0, 6.2, 7.5 and 9.0, respectively. The conditions were optimized and the best solvent for operation was found to be acetonitrile at *λ*_ex_ at 340 nm. Folic acid was successfully estimated in tablet dosage form, urine and serum in the concentration range of 2.49 × 10^−9^ to 1.28 × 10^−6^ mol L^−1^. Progesterone, testosterone and vitamin D_3_ were also assessed in serum samples using the same optimal conditions within concentration ranges of 5 × 10^−9^ to 1.9 × 10^−6^, 5 × 10^−9^ to 2.8 × 10^−6^ and 5 × 10^−9^ to 4.2 × 10^−6^ mol L^−1^, respectively. The proposed luminescence method was validated in accordance with ICH guidelines and found to be accurate, precise, and specific and free from any interference at the working pH for each analyte. The cost effectiveness and applicability of the method make it a good choice for routine analysis of the four compounds and early diagnosis of chronic diseases associated with abnormalities in their physiological levels.

## Abstract 2

An innovative, simple and cost effective Tb^3+^–simvastatin photo probe was designed and used as a core for a spectrofluorometric approach to sensitively determine four vital biological compounds in different matrices.

## Graphical abstract

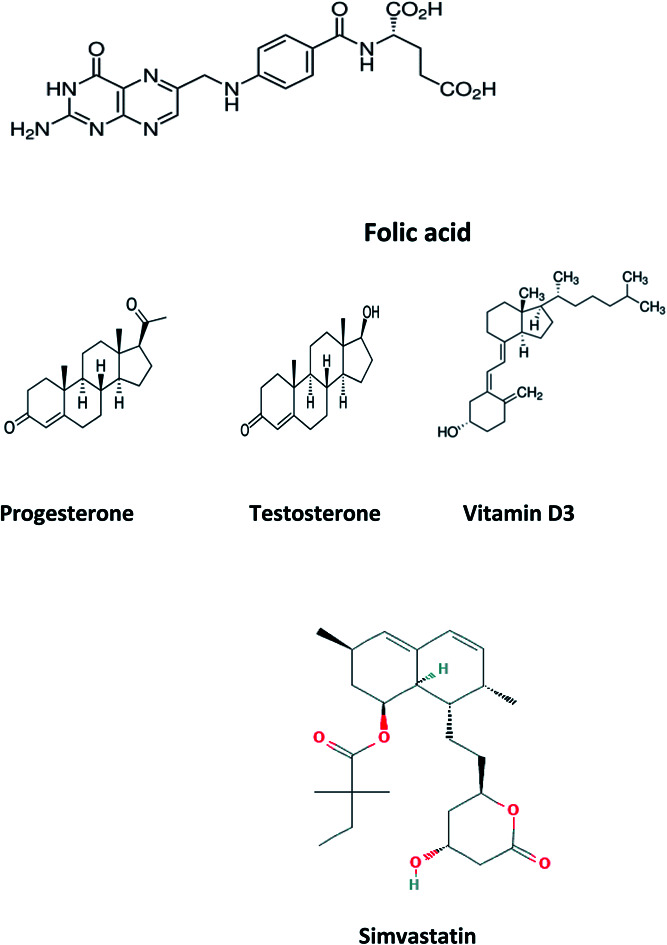

## 1. Introduction

**Fig. 1:**
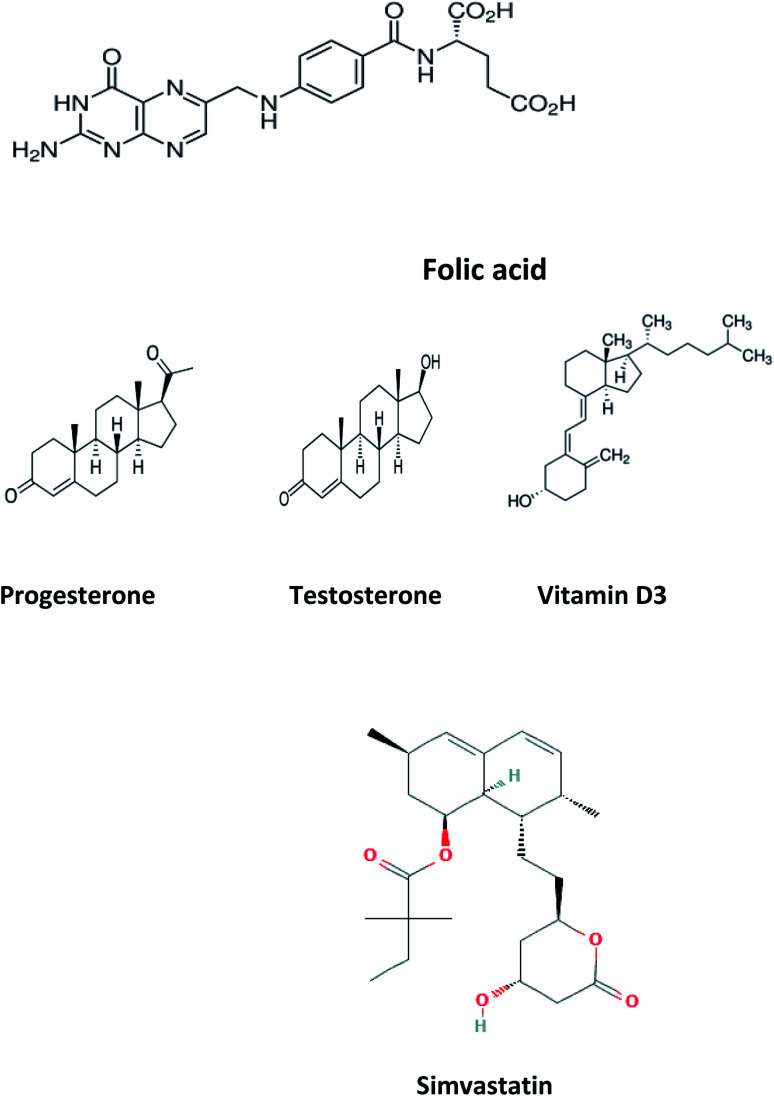
Chemical structure of folic acid, progesterone, testosterone, vitamin D_3_ and simvastatin.

Folic acid (FCA), vitamin B9, is a water-soluble vitamin^1^ found naturally in various types of foods as legumes, leafy green vegetables, wheat germs, beets, broccoli, citrus fruits, fermented products, beef liver and eggs. FCA is an essential supplement for pregnant women in the first trimester to avoid birth abnormalities including congenital heart diseases and neural tube defects and autism.^2^ FCA is essential for DNA and RNA production and amino acid metabolism.^3^ Untreated deficiency of FCA is linked with different health problems, including neurological and psychological manifestations like psychosis, depression, insomnia, and Alzheimer's disease, increase risk of cancer and osteoporosis.^4^ Elevated levels of homocysteine, a biomarker for arteriosclerosis, is also associated with FCA deficiency. Other symptoms include poor cognitive performance, hearing loss and other symptoms including fatigue, heart palpitations, shortness of breath, hair and skin discoloration, mouth sores, and swollen tongue.^5^ Different analytical techniques were reported in the literature for FCA determination in dosage form, dietary supplements, beverages and biological samples including spectroscopy^6^ and chromatography^7^ and electrochemistry.^8^ The chemical structure of folic acid is presented in Fig. 1.

Progesterone (PGS) is a member of progestogen steroid hormones group, secreted mainly during the menstrual cycle by the corpus luteum preparing the body for conception in case of ova fertilization.^9^ PGS is used in oral contraception either in single form or combined with estrogen and as hormonal replacement therapy to alleviate menopause symptoms. Low levels of progesterone may lead to abnormal bleeding during menstruation, premature labor and miscarriage during pregnancy and considered as sign for poly-cystic ovarian syndrome. While PGS elevated level may increase the risk of breast cancer development and marker for adrenal hyperplasia. PGS concentrations was recently estimated using different analytical approaches; spectroscopic,^10^ chromatographic,^11^ electrochemical methods^12^ and immunological assay.^13^ The chemical structure of PGS is presented in Fig. 1.

Testosterone (TST), an anabolic steroid, is the primary male sex hormone where it regulates RBCs production, libido, fertility, spermatogenesis, fat distribution, bone and muscle mass.^14^ Imbalance in levels TST may can cause serious body dysfunctions where diminished levels have an inverse impact sexual drive, erection, sperm count and muscle strength. Abnormal high TST levels may trigger early puberty in males and menstrual irregularities and baldness in females.^15^ TST could also be as a medication to replenish its insufficiency, manage breast cancer in women and enhance physique and performance, for instance in athletes.^16^ Its concentration in different matrices as plasma, serum, saliva was recently measured through spectroscopic^17^ chromatographic^18^ electrochemical methods^19^ and capillary electrophoresis.^20^Fig. 1 shows the structure of TST.

Vitamin D_3_, one of fat-soluble vitamins, is naturally found in different types of foods as oily or fatty fish, dairy products, beefy liver and egg yolk and synthetized endogenously in human body upon exposure to sun. Vit. D_3_ is converted to its active form through two successive hydroxylation steps forming calcidiol (25-hydroxyvitamin D) in liver followed by calcitriol (1,25-dihydroxyvitamin D) in kidney. It has a major role in regulation concentration of phosphate and calcium in serum and essential in bone remodeling and growth.^21^ It is also used to improve the cognitive functions and in treatment of specific type of psoriasis. In addition, it contributed to the management of Covid-19 by reducing the cytokine storms and thrombotic episodes associated with the infection.^22^ The deficiency of Vit. D may lead to serious conditions as rickets and osteomalacia in young and adults, respectively.^23^ Low levels of Vit. D is also associated with increased risk of colon and pancreatic cancer respiratory acute infections.^24^ On the other hand, the excessive intake of Vit. D may increase the levels of calcium both in soft tissues (calcinosis) and blood (hypercalcemia). To evaluate the status of Vit. D in human body, calcidiol level in blood is used as best indicator. The chemical structure of Vit. D is displayed in Fig. 1. In the last decade, quantification of Vit. D and/or its metabolites was established through chemiluminescent assay,^25^ chromatography.^26^ The reported methods showed relatively high limits of detection which restricts their practical applications. Moreover, the measurement of low concentrations of folic acid, progesterone, testosterone and vitamin D_3_ in biological samples along with interference from some biomolecules such as uric acid (UA), ascorbic acid (AA), and different hormones requires to efficiently improve the sensitivity of chromatographic methods and the electrochemical sensors for practical applications. Therefore, developing a simple method for accurate determination of folic acid, progesterone, testosterone and vitamin D_3_ in the presence of each other in the same sample is still of great significance. Today, the research field in which the lanthanide complexes were used as biosensors has a great interest.^27–42^ Luminescent optical biosensor Tb(simvastatin)_3_ (Tb–SIM) complex embedded in PEG matrix have many advantages over the mentioned traditional methods. Terbium ion has sharp and precise emission bands in green light region. The terbium ion is used as photo probe for many analytes with a high selectivity depends on the excitation wavelength of terbium–analyte complex, pH and the type of solvent of the test solution. Doping of the optical sensors in the polymer matrix increases its stability and durability.^30–36^ The sensor can provide a constant signal response for two years, which makes it 24-fold better balance compared to the lifetime warranted for the chromatographic and electrochemical methods. The source of error of the present work eliminated as it more stables for a long time; it gives a low standard deviation value. The higher stability of the current sensor can be attributed to the doping of the optical sensor in the polymer matrix.

## 2. Experimental

### 2.1. Instrumentation

A double beam UV-Visible spectrophotometer (PerkinElmer Lambda 25), fluorescence Spectrometer (Thermo Scientific Lumina, Meslo-PN; 222-263000). pH meter (Jenway; 33300)

### 2.2. Materials and reagents

Pure folic acid standard was kindly supplied by the National Organization for Drug control and Research (Giza, Egypt). Pharmaceutical preparation of folic acid tablets dosage form labelled to contain 500 μg manufactured by Mepaco-Medifood (Arab Company for Pharmaceutical and Medicinal plants, Egypt) was purchased from community pharmacy in the Egyptian market. Progesterone, testosterone, vitamin D_3_, solvents including ethanol, acetonitrile, dimethylformamide (DMF), chloroform and dimethyl sulfoxide (DMSO) were purchased from Sigma Aldrich. Analytical grade ammonium hydroxide (NH_4_OH), hydrochloric acid (HCl), Tb (NO_3_)_3_·5H_2_O, simvastatin and polyethylene glycol (PEG) were purchased from Sigma Aldrich.

The Human real samples were gathered from both Ain Shams Specialized and Teaching New Al-Kasr-El-Aini Hospitals, Cairo, Egypt in accordance with the approved protocol of World Health Organization (WHO) for the collection of human specimens and the use of the clinically related information and data for the purpose of research. The patients approved and were all consented before using their samples.

### 2.3. Preparation of standard solutions

Stock solutions of Tb (NO_3_)_3_·5H_2_O and simvastatin; were prepared separately by accurately weighing and transferring 0.11 g and 0.039 g, respectively of their authentic pure forms into separate 25 mL volumetric flasks by the aid of the least amount of ethanol till dissolution and completing the volume with the same solvent to obtain final concentration of (10^−2^ mol L^−1^) for each of them.

Tb^3+^–simvastatin complex solution; was prepared by mixing 0.1 mL of Tb(NO_3_)_3_ stock solution with 0.3 mL of simvastatin (Fig. 1) stock solution in 10 mL volumetric flask and completing the volume to the mark with acetonitrile.

For the four compounds under study, all stock solutions were separately prepared in 10 mL volumetric flasks in concentration of solution (10^−2^ mol L^−1^). This was achieved by dissolving 0.044 g of FCA in least amount of DMF and then completing the volume to the mark using acetonitrile. For PGS, TST and Vit. D 0.031 g, 0.0288 g, 0.033 g, were dissolved, respectively in small amount of ethanol and then volume was diluted to the mark with acetonitrile. Further dilutions for the stock solutions using acetonitrile were performed to obtain working solutions with concentrations of 1.0 × 10^−4^ to 1.0 × 10^−9^ mol L^−1^ of FCA, PGS, TST and Vit. D.

0.1 mol L^−1^ of NH_4_OH and HCl were used to adjust the pH to 9.0, 5.0, 6.2, 7.5 for FCA, PGS, TST and Vit. D, respectively. All the prepared solutions should be kept at low temperature (2–8 °C) to remain stable.

### 2.4. Preparation of FCA pharmaceutical dosage form solution

Ten tablets of Folic acid® 500 μg were weighed and grinded into fine homogenous powder. The average weight of one tablet was calculated and dissolved in few mL of DMF and sonicated for 20 minutes. The solution was then filtered using whattman filter papers (12 mm) into 10 mL volumetric flask to obtain final concentration of FCA equivalent to 1.1 × 10^−3^ mol L^−1^. Further dilution was performed to obtain different solutions with concentration range of (1.0 × 10^−4^ to 1.0 × 10^−7^ mol L^−1^) was prepared by appropriate dilution with acetonitrile.

### 2.5. Preparation of urine sample spiked with FCA

The urine sample was collected from a healthy volunteer who didn't administer any previous medications, it was then manipulated in the lab as follows; 10 mL of the collected urine sample were centrifuged at 4000 rpm for 15 min to remove all interferants including crystals, salts, pus and red blood cells. 1.0 mL of urine was spiked with 1.0 mL of previously prepared drug solution with concentration of 1.0 × 10^−6^ mol L^−1^ and completed by acetonitrile to the mark in 10 mL measuring flask.

### 2.6. Preparation serum samples spiked with FCA, PGS, TST and Vit. D_3_

A 1.0 mL of samples of blood collected from healthy volunteers was centrifuged for 15 min at 4000 rpm to remove proteins. 0.1 mL of the serum sample was added to 1.0 mL of each drug working solution of concentration 1.0 × 10^−6^ mol L^−1^ and the volume was complete to 10 mL by acetonitrile to obtain 1.0 × 10^−7^ mol L^−1^ for each drug in four separate 10 mL measuring flasks.

### 2.7. Preparation of Tb–SIM biosensor embedded in PEG

Tb–SIM complex was prepared in the solid state by mixing an equal volume of 1.0 × 10^−4^ mol L^−1^ Tb ion and 3.0 × 10^−4^ mol L^−1^ simvastatin in ethanol, then evaporation near the dryness of the solution, a pale pink solid was obtained after cooling in air. The thin film was prepared by dissolving 0.1 g of the solidified and seamless complex in 3 mL ethanol and then adding 10 mL of viscose freshly prepared PEG with stirring for about one hour until a homogenous solution was obtained. A thin film was fabricated by spin-coating on a small quartz slide (width 8.5 mm, height 25 mm) to quick fit in the cuvette of the spectrofluorometer.

### 2.8. Recommended procedure

An appropriate volume (100 μL) of various standard concentrations of folic acid, progesterone, testosterone and vitamin D_3_ should be diluted to 3 mL with acetonitrile. The dilute solution was mixed with a thin film of biosensing Tb–SIM doped in PEG matrix in the quartz cell of a spectrofluorometer. The luminescence spectra were recorded at the excitation wavelength *λ*_ex_ = 340 nm. After each measurement, the optical sensor was washed with acetonitrile, and the calibration curve was built by applying the Stern's Volmer equation by plotting (*F*/*F*_o_) the at *λ*_em_ = 545 nm on the *y*-axis *versus* the folic acid, progesterone, testosterone and vitamin D_3_ concentration in mol L^−1^ on the *x*-axis.

### 2.9. Determination of FCA in tablet dosage form

The tablet dosage form solutions previously prepared under (2.4) were analyzed using the following procedures: in the spectrofluorometer cell, 1.0 mL of the tablet solutions was separately added followed by the 1.5 mL of acetonitrile in presence of the biosensor film. After mixing, the obtained solutions were scanned, and luminescence spectra were recorded at *λ*_ex_/*λ*_em_ = 340/545 nm. The concentrations of the real samples were calculated using corresponding regression equation.

### 2.10. Determination of FCA in spiked urine samples

The luminescence spectra of the previously prepared spiked urine samples as detailed under (2.5) were scanned at *λ*_ex_/*λ*_em_ = 340/545 nm and the concentration of spiked FCA was determined using the corresponding regression equation adopting the standard addition technique.

### 2.11. Determination of FCA, progesterone, testosterone and vitamin D_3_ in serum samples

The luminescence spectra of the serum samples previously prepared as described under (2.6) were measured adopting the same procedures followed under (3.2). The concentrations of each real sample were calculated using corresponding regression equation.

## 3. Result and discussion

### 3.1. General features of absorption and emission spectra of Tb–SIM complex

Owing to the f–f transition forbiddance of trivalent ion (Tb^3+^), there is a restriction to directly absorb light which could be overcome through the antenna effect *via* the coupling between Tb^3+^ and a prominently absorbing organic ligand leading to efficient energy transfer and light absorption processes. Regarding the proposed photo probe, Tb^3+^ is surrounded covalently by 3 molecules of simvastatin ligand responsible for efficient absorption of light and transfer of energy to populate ^5^D_4_ state of Tb^3+^.^43^

The emission of the formed complex Tb–SIM exhibited four specific and intense bands because of the ^5^D_4_–^7^F_*J*_ transitions (*J* = 6, 5, 4 and 3).^44^

### 3.2. Absorption and emission spectra

**Fig. 2:**
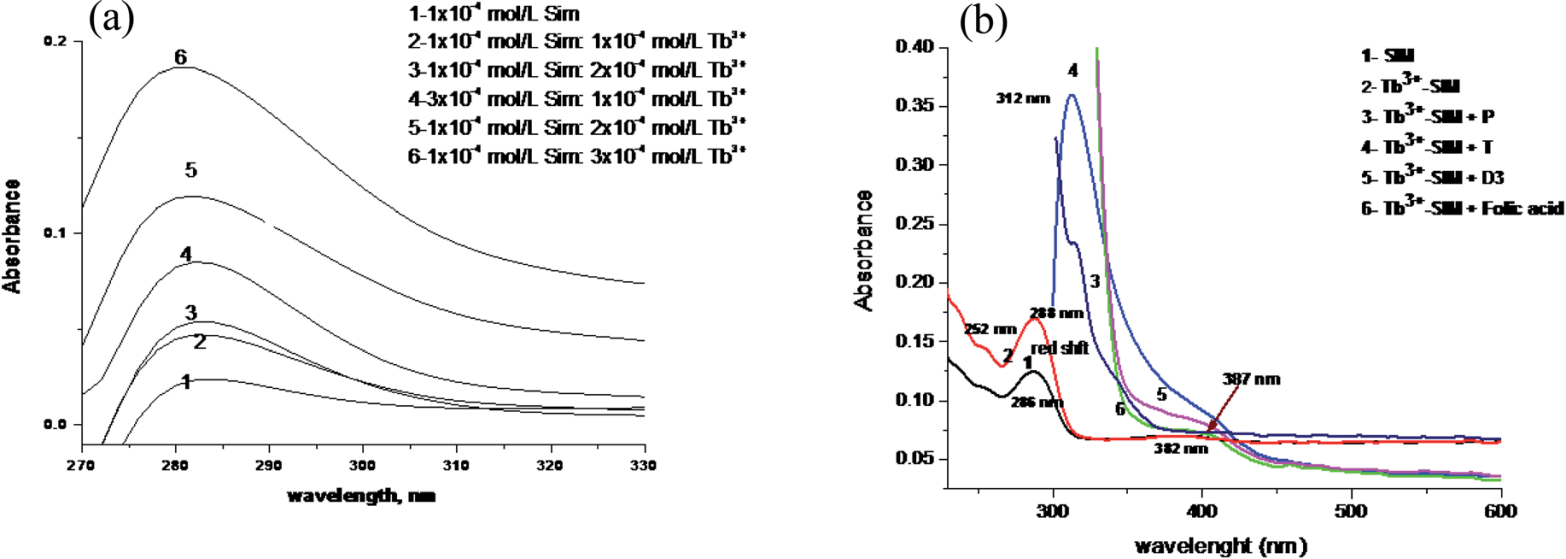
(a) The absorption spectra of different molar ratios of 1 × 10^−4^ M Tb(NO_3_)·6H_2_O with 1 × 10^−4^ M of simvastatin in acetonitrile. (b) The absorption spectrum of (1) simvastatin, (2) Tb^3+^–simvastatin complex (Tb^3+^–SIM), (3) Tb^3+^–simvastatin–progesterone (P), (4) Tb^3+^–simvastatin–testosterone (T), (5) Tb^3+^–simvastatin–vitamin D_3_ (Vit D_3_), (6) Tb^3+^–simvastatin–folic acid.

The absorption spectra of Tb (NO_3_)_3_, simvastatin and Tb^3+^–simvastatin complex are shown in Fig. 2a. A red shift by 7 nm and the absorbance value is enhanced denoting that simvastatin could form a stable complex with Tb^3+^. The absorption spectra of FCA, PGS, TST and Vit. D_3_ were scanned alone and in the presence of the optical sensor are shown in Fig. 2b.

**Fig. 3:**
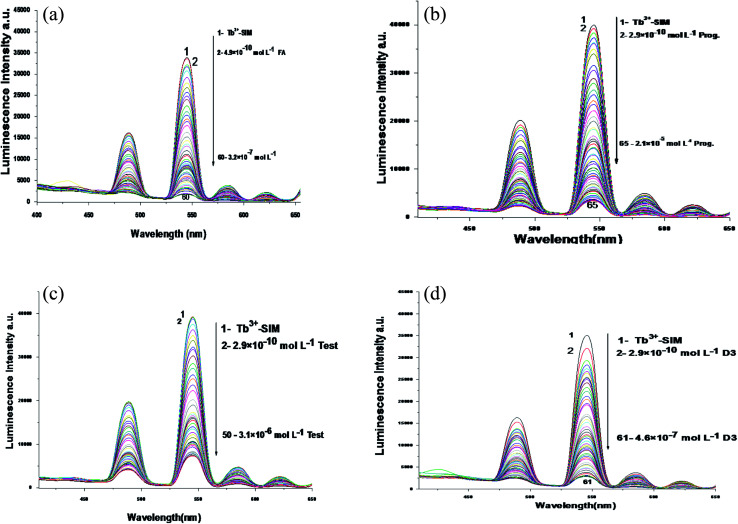
(a) The emission spectra of Tb^3+^–SIM complex at *λ*_ex_ = 340 nm and pH 5.0 in presence of different folic acid concentrations using acetonitrile as a solvent. (b) The emission spectra of Tb^3+^–SIM complex at *λ*_ex_ = 340 nm and pH 6.2 in presence of different progesterone concentrations using acetonitrile as a solvent. (c) The emission spectra of Tb^3+^–SIM complex at *λ*_ex_ = 340 nm and pH 7.5 in presence of different testosterone concentrations using acetonitrile as a solvent. (d) The emission spectra of Tb^3+^–SIM complex at *λ*_ex_ = 340 nm and pH 9.0 in presence of different vitamin D3 concentrations using acetonitrile as a solvent.

The emission spectra of Tb^3+^–SIM complex after adding different concentrations of FCA, PGS, TST and Vit. D_3_ using acetonitrile as solvent are shown in Fig. 3a–d, respectively. The characteristic electrical emission band of Tb^3+^ exhibited at *λ*_em_ 545 nm was quenched due to energy transfer from the optical sensor to FCA, PGS, TST and Vit. D_3_.

### 3.3. Experimental variables

#### 3.3.1. Tb^3+^ and simvastatin amounts

**Fig. 4:**
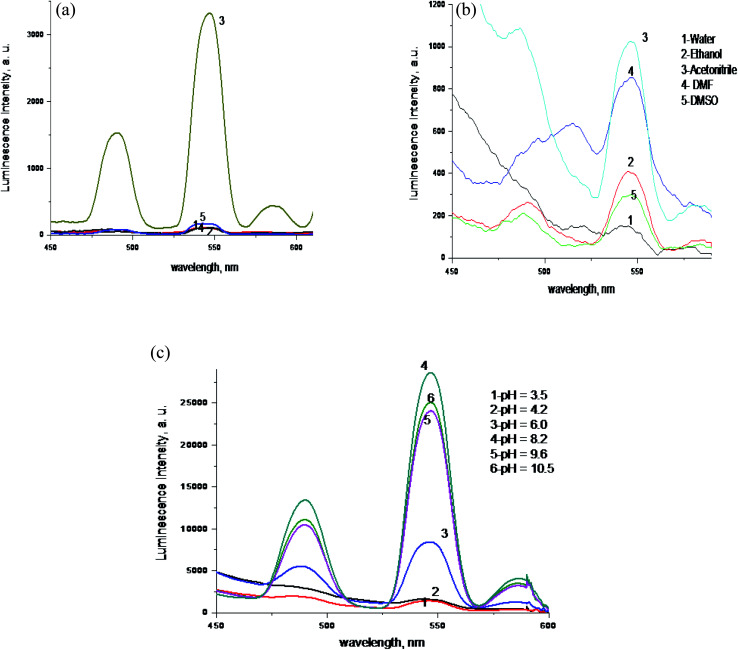
(a) Luminescence spectra of 1.0 × 10^−4^ mol L^−1^ Tb^3+^ with 1.0 × 10^−4^ mol L^−1^ of simvastatin in different molar ratio; 1 – (1Tb : SIM), 2 – (1Tb : 2SIM), 3 – (1Tb : 3SIM), 4 – (2Tb : 1SIM), 5 – (3Tb : 1SIM) in acetonitrile at *λ*_ex_ = 340 nm. (b) Emission spectra of Tb^3+^–SIM optical sensor in different solvents at *λ*_ex_ = 340 nm and pH 8.2. (c) Emission spectra of Tb^3+^–SIM optical sensor in acetonitrile at *λ*_ex_ = 340 nm and different pHs.

The Tb^3+^–simvastatin complex was formed in ratio 1 M: 3 L indicating that the metal coordinates to the ligand at different sites of coordination not *via* oxygen only, Fig. 4a.

#### 3.3.2. Solvent effect

The intensity of luminescence of solutions containing Tb^3+^ (1.0 × 10^−4^ mol L^−1^) and simvastatin (3.0 × 10^−4^ mol L^−1^) was investigated in different solvents and the results revealed that maximum enhancement was noticed in acetonitrile as presented in Fig. 4b. Solvents with hydroxyl group as ethanol diminishes the luminescence intensity due to transfer of vibrational energy to molecules of solvents.^45–52^

#### 3.3.3. pH effect

The medium pH has a significant influence on the luminescence intensity of the formed Tb^3+^–simvastatin complex. Solutions of NH_4_OH and HCl, both 0.1 mol L^−1^ were used for pH adjustment. The highest luminescent intensity at *λ*_em_ 545 nm was observed at pH = 8.2 as shown in Fig. 4c.^53–60^

### 3.4. Mechanism of emission quenching

Upon adding different concentrations of FCA, PGS, TST and Vit. D_3_ to the Tb–SIM photo probe a notified quenching in its luminescent intensity occurs owing to the approach of the analytes under study and formation of H-bond between the hydroxyl group in both of TST and Vit. D_3_, carboxylic group in FCA and enol group in PGS with the SIM. The formation of H-bonding lead to the depression or decrease in the transfer of energy to the Tb^3+^ ion and consequently the luminescence intensity is significantly quenched.

The pH effect on the luminescence intensity after the addition of the studied analytes to the proposed photoprobe was studied and the luminescence quenching was observed at pH 5.0, 6.2, 7.5 and 9.0 for FCA, PGS, TST and Vit. D_3_ respectively.

## 4. Analytical performance^61^

### 4.1. Linearity

**Fig. 5:**
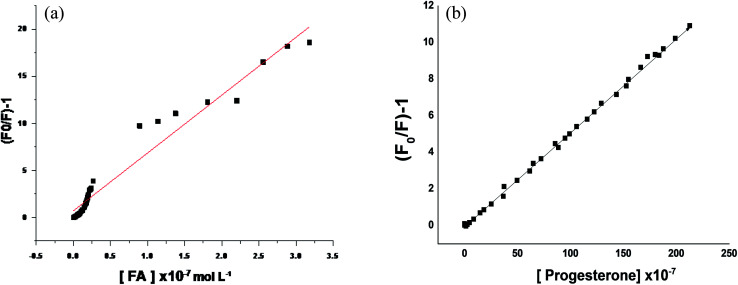
(a) Stern–Volmer plot (*F*_o_/*F*) − 1 against corresponding concentrations of folic acid. (b) Stern–Volmer plot (*F*_o_/*F*) − 1 against corresponding concentrations of progesterone.

Correlations between the luminescence of emission intensity of optical sensor at *λ*_em_ 545 nm and FCA, PGS, TST and Vit. D_3_ within concentration ranges of (2.4 × 10^−9^ to 1.28 × 10^−6^), (5 × 10^−9^ to 1.9 × 10^−6^), (5 × 10^−9^ to 2.8 × 10^−6^) and (5 × 10^−9^ to 4.2 × 10^−6^) mol L^−1^ respectively were found to be linear as presented in respective calibration graphs, Fig. 5a and b obtained by applying the Stern–Völmer plot.

The critical concentration of FCA, PGS, TST and Vit. D_3_ values are (3.31, 3.1, 2.2, 1.3) and (0.005 × 10^−7^ to 3.18 × 10^−7^, 2.49 × 10^−10^ to 2.12 × 10^−5^, 2.4 × 10^−10^ to 3.18 × 10^−6^, 4.9 × 10^−10^ to 4.8 × 10^−7^) mol L^−1^ respectively. The distance between the cited compounds and the ionophore is 3.36 Å indicating the electron transfer mechanism of quenching.

The regression equations were computed and the regression parameters in addition the LOD and LOQ were calculated, and results were presented in Table 1.

**Table 1:** Validation sheet and parameters of the regression equations of the proposed optical sensor

Parameter	Folic acid	Progesterone	Testosterone	Vitamin D_3_
*λ*_em_ (nm)	545			
Linearity (mol L^−1^)	1.28 × 10^−6^ to 2.49 × 10^−9^	1.9 × 10^−6^ to 5 × 10^−9^	2.8 × 10^−6^ to 5 × 10^−9^	4.2 × 10^−6^ to 5 × 10^−9^
LOD (mol L^−1^)	1.99 × 10^−9^	1.5 × 10^−9^	3.02 × 10^−9^	1.59 × 10^−9^
LOQ (mol L^−1^)	5.94 × 10^−9^	4.5 × 10^−9^	9.0 × 10^−9^	2.8 × 10^−9^
Regression equation	(*Y* = *a* + *bX*)^a^			
Intercept (*a*)	0.32	47.2	105	84.5
Slope (*b*)	3.31	3.1	2.2	1.30
Standard deviation	0.04	15.5	20.5	6.40
Variance (*S*^2^)	0.0016	240.25	420.25	4.90
Regression coefficient (*r*)	0.99	0.99	0.99	0.99

a Where *Y*: intensity of luminescence, *X*: analyte concentration (mol L^−1^), *a*: intercept and *b*: slope.

### 4.2. Accuracy and precision

The accuracy of the developed method was further investigated *via* applying the standard addition technique and calculating the recovery%. Assessing the obtained recovery was performed through determination of agreement extent between the measured and actual added standard concentration of analyte. All assays were repeated 3 times within the same day and different days to assess the repeatability and intermediate precision, respectively. Three different levels of the analyte concentrations were used in the assays and the results were summarized and presented in (Table 2).

**Table 2:** Evaluation of repeatability and intermediate precision of the proposed optical method^a^

Sample	Concentration taken (×10^−7^ mol L^−1^)	Repeatability			Intermediate precision		
		Average found ± CL^b^	% RE^c^	% RSD^d^	Drug average found ± CL	% RE	% RSD
Progesterone in serum	1.0	1.03 ± 0.13	3.0	3.39	1.06 ± 0.11	6.0	2.13
	2.0	1.95 ± 0.18	2.5	2.33	2.05 ± 0.17	2.5	3.12
	4.0	4.19 ± 0.24	4.75	2.99	4.20 ± 0.23	5.0	2.11
Testosterone in serum	1.0	1.11 ± 0.13	11.0	3.46	0.99 ± 0.11	1.00	3.11
	2.0	2.02 ± 0.18	1.00	2.41	2.04 ± 0.16	2.00	2.06
	4.0	3.89 ± 0.26	2.75	2.95	4.13 ± 0.21	3.25	3.02
Vitamin D_3_ in serum	1.0	1.06 ± 0.23	6.00	2.22	1.09 ± 0.21	9.00	2.01
	2.0	2.05 ± 0.28	2.50	2.26	2.14 ± 0.36	7.00	4.35
	4.0	4.19 ± 0.48	4.75	2.25	4.23 ± 0.31	5.75	2.51
Tablet, 500 μg of folic acid MEPACO	3.0	3.04 ± 0.024	1.33	0.33	3.07 ± 0.052	2.33	0.68
	6.0	5.99 ± 0.050	0.16	0.35	6.08 ± 0.070	1.33	0.47
	9.0	8.96 ± 0.025	0.33	0.11	9.09 ± 0.062	1.00	0.28
Folic acid in serum	4.0	3.98 ± 0.20	0.50	0.38	4.08 ± 0.038	2.00	0.37
	6.0	5.98 ± 0.15	0.33	0.61	6.09 ± 0.080	1.50	0.53
	9.0	9.01 ± 0.22	0.22	0.33	9.06 ± 0.062	0.67	0.28
Folic acid in urine	4.0	3.99 ± 0.20	0.50	0.10	4.06 ± 0.043	2.03	0.32
	6.0	5.99 ± 0.15	0.33	0.66	6.07 ± 0.070	1.54	0.51
	9.0	8.99 ± 0.22	0.22	0.44	9.04 ± 0.066	0.74	0.38

a *n* = 3.

b CL: confidence limits (supplementary material).

c % RE: percent relative error.

d RSD: relative standard deviation.

### 4.3. Selectivity

The selectivity of the proposed method was investigated through analyzing placebo blank and synthetically prepared mixtures. All possible interfering inactive compounds were used to prepare a placebo containing; 50 mg calcium carbonate, 20 mg calcium dihydrogen orthophosphate, 30 mg lactose, 100 mg magnesium stearate, 40 methyl cellulose, 70 mg sodium alginate, 300 mg starch and 250 mg Talc. Extraction was performed using water and the solution was manipulated as detailed under 2.4. A suitable aliquot of the obtained solution was analyzed after the addition of the optical sensor Tb^3+^–simvastatin, and the luminescence spectra were recorded at *λ*_ex_/*λ*_em_ = 340/545 nm following the optimized conditions.

The validity and selectivity were further assessed in presence of some proteins and hormones that may interfere as cortisol, Thyroid stimulating hormone, norepinephrine, dopamine and albumin within concentration range of 0.08 g L^−1^. The interference of 0.0.06 g L^−1^ urea, 0.08 g L^−1^ glucose, uric acid and folic acid was also studied, and the resulting data revealed that there was no significant effect on the observed luminescence activity of the proposed photo probe under optimized conditions.

In addition, the proposed optical probe was successfully applied for selective determination of FCA, PGS, TST and Vit. D_3_ either as single or in combination in synthetically prepared mixtures. Four synthetic mixtures were prepared by adding different concentrations of FCA, PGS, TST and Vit. D_3_ within their linearity range in 4 similar sets of 10 mL volumetric flasks containing 1.0 mL of the serum sample as mentioned under 2.6.

The pH of the first set was adjusted to 5.0 for selective determination of FCA in presence of PGS, TST and Vit. D_3_, the pH of the second set was adjusted to 6.2 for the determination of PGS in presence of FCA, TST and Vit. D_3_, the pH of the third set was adjusted to 7.5 for determination of TST in presence of FCA, PGS, and Vit D_3_, finally the pH of the fourth set was adjusted to 9 for determination of Vit D_3_ in presence of FCA, PGS, TST and the volume was completed with acetonitrile for the four sets. Thus, each mixture was prepared 4 times but at different pH (5.0, 6.2, 7.5 and 9.0) for selective estimation of FCA, PGS, TST and Vit. D_3_, respectively. Each solution was in triplicates and yielded recovery% of 99.60 ± 0.47,100.8 ± 2.10, 99.4 ± 2.60 and 101.9 ± 2.20 for FCA, PGS, TST and Vit. D_3_, respectively.

**Fig. 6:**
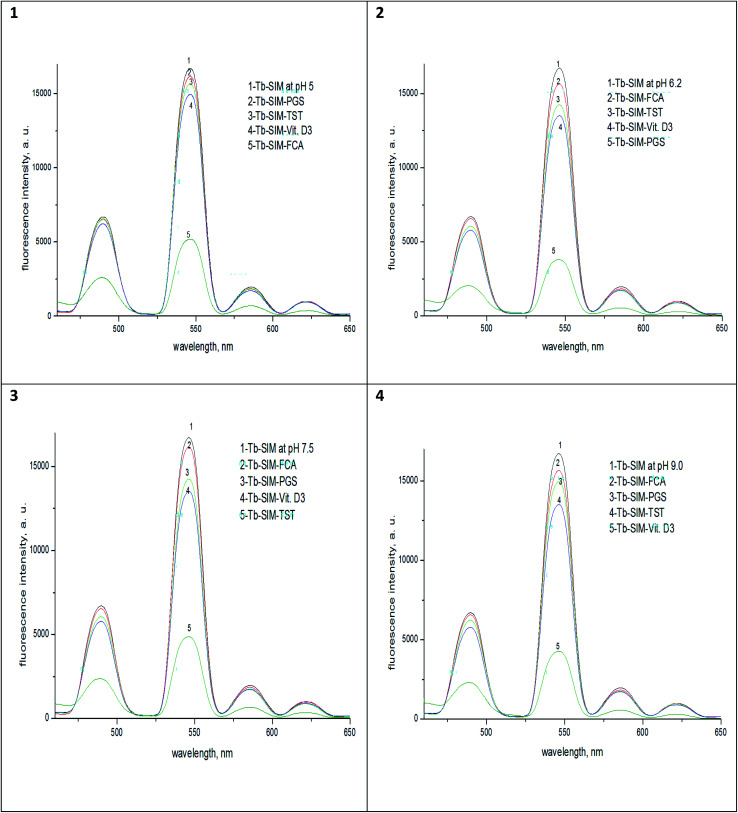
The luminescence spectra of the complexes Tb–SIM, Tb–SIM–FCA, Tb–SIM–PGS, Tb–SIM–TST and Tb–SIM–Vit-D_3_ at *λ*_ex_ = 340 nm and different pHs; (1) 5.0, (2) 6.2, (3)7.5 and (4) 9.0.

Results in Fig. 6 show that the luminescence of Tb^3+^–SIM complex in its second coordination sphere in which the quaternary mixture of FCA, PGS, TST and Vit. D_3_ is quite sensitive to four variant sets of pHs. For Tb^3+^–SIM–FCA, *λ*_ex_ = 340 and pH 5.0, give the more quenching of luminescence intensity of Tb^3+^–SIM while that for Tb^3+^–SIM–PGS was of *λ*_ex_ = 340 and pH 6.2 and that for Tb^3+^–SIM–TST was of *λ*_ex_ = 340 and pH 7.5, and that for Tb^3+^–SIM–Vit-D_3_ was of *λ*_ex_ = 340 and pH 9.0. Thus, a dual-controlled luminescence of smoothly dynamic reversibility is achieved and a reversible on/off switchable Tb^3+^ emission of one system was observed by tuning its optimal values of pH to the optimal ones of the second and so on for the third and fourth. By this dual controlled luminescence, the quaternary mixture of FCA, PGS, TST and Vit. D3 was simultaneously resolved with average error <3.5%.

Also, the data obtained upon assaying single PGS, TST and Vit. D_3_ separately in serum sample and FCA in serum, urine and dosage form, without any interference from inactive excipients, were processed and results were tabulated as shown in Table 3. The results of the proposed method were comparable to that obtained from the reference chromatographic methods mentioned in the British pharmacopeia.^62^ The limitations of the proposed method in real samples in which a hormones and proteins are existed. These biological molecules contain OH, NH and SH groups may make an interference with the analytes at different pHs.

**Table 3:** Determination of folic acid, progesterone, testosterone and vitamin D_3_ samples using Tb–SIM optical sensor

Sample	Added (× 10^−7^ mol L^−1^)	Found (× 10^−7^ mol L^−1^)	Average^a^ (× 10^−7^ mol L^−1^)	Average recovery ± % R.S.D	B·P. (LC)
Progesterone serum sample	3.5	3.52, 3.48, 357	3.52	100.2 ± 2.1	98.6 ± 0.5
	7.0	6.97, 7.05, 7.03	7.01		
	9.5	9.55, 9.46, 9.46	9.49		
Testosterone serum sample	3.5	3.49, 3.53, 3.52	3.51	99.6 ± 2.5	99.2 ± 0.6
	7.0	6.95, 6.99, 6.98	6.97		
	9.5	9.51, 9.45, 9.48	9.48		
Vitamin D_3_ serum sample	3.5	3.39, 3.43, 3.62	3.48	103.1 ± 2.9	99.4 ± 0.5
	7.0	6.85, 6.89, 6.88	6.87		
	9.5	9.41, 9.55, 9.58	9.51		
Tablet, 500 μg of folic acid MEPACO-MEDIFOOD	3.0	3.04, 3.05, 3.03	3.04	101.33 ± 0.33	99.8 ± 0.055
	6.0	6.02, 5.98, 5.99	5.99	99.83 ± 0.35	
	9.0	8.97, 8.96, 8.95	8.96	99.66 ± 0.11	
Folic acid serum sample	4.0	3.98, 3.97, 4.00	3.98	99.5 ± 0.38	99.6 ± 0.050
	6.0	6.01, 5.97, 5.95	5.98	99.66 ± 0.61	
	9.0	8.98, 8.95, 9.01	9.01	99.77 ± 0.33	
Folic acid urine sample	4.0	3.99, 3.97, 4.01	3.99	99.75 ± 0.10	99.5 ± 0.050
	6.0	6.02, 5.98, 5.99	5.99	99.83 ± 0.66	
	9.0	8.99, 8.97, 9.00	8.99	99.88 ± 0.44	

a Average of nine measurements.

### 4.4. Comparison with previously reported methods

The results obtained from the proposed spectrofluorometric technique was compared with obtained from other previously reported methods assuring the applicability, accuracy, and precision of the proposed method as presented in Table 4,^8,9,14,15,17,20,21,29–33,63,64^

**Table 4:** Comparison of proposed optical luminescent technique *versus* some previously reported methods for estimation of progesterone, testosterone, vitamin D_3_ and folic acid

Analyte	Methods	Linearity	Limit of detection	References
Progesterone	HPLC-MS-MS	0.2–50 ng mL^−1^	0.2 ng mL^−1^	18
	Microfluidic immunosensor system	0.5–12.5 ng mL^−1^	0.2 ng mL^−1^	21
	Enzyme-linked fluorescence assay	3–40.0 ng mL^−1^	—	22
	Spectrofluorometric using Tb^3+^–SIM	1.9 × 10^−6^ to 5 × 10^−9^ mol L^−1^	1.49 × 10^−9^ mol L^−1^	
Testosterone	HPLC in plasma	1.6–400 ng mL^−1^	1.6 ng mL^−1^	32
	HPLC in serum	1–20 ng mL^−1^	0.4	33
	HPLC in urine	10–500 ng mL^−1^	1 ng mL^−1^	30
	HPLC in dosage form	50–200 μg mL^−1^	5 μg mL^−1^	31
	HPLC in urine	2–300 ng mL^−1^	2 ng mL^−1^	29
	Spectrofluorometric using Tb^3+^–SIM	2.8 × 10^−6^ to 5 × 10^−9^ mol L^−1^	3.1 × 10^−9^ mol L^−1^	
Vitamin D_3_	HPLC	15–200 nmol L^−1^	3 nmol L^−1^	62
	LC-MS/MS	3.5 to 75 ng mL^−1^	14 ng mL^−1^	63
	HPLC-APCI-MS	5–400 nmol L^−1^	1–4 nmol L^−1^	64
	Spectrofluorometric using Tb^3+^–SIM	4.2 × 10^−6^ to 5 × 10^−9^ mol L^−1^	1.6 × 10^−9^ mol L^−1^	
Folic acid	LC-MS/MS	4.5 × 10^−8^ to 5 × 10^−10^ mol L^−1^	5 × 10^−10^ mol L^−1^	15
	Chemiluminometric and fluorimetric determination	114–6.0 μg mL^−1^, 1.10–0.022 μg mL^−1^	2.0 μg mL^−1^, 0.002 μg mL^−1^	8
	Chemiluminescence	8 × 10^−7^ to 6 × 10^−9^ mol L^−1^	6 × 10^−103^ mol L^−1^	9
	HPLC method	2500 to 50 μg mL^−1^	1.3 ng mL^−1^	14
	Spectrofluorometric method: using Tb^3+^–SIM	1.28 × 10^−6^ to 2.49 × 10^−9^ mol L^−1^	1.99 × 10^−9^ mol L^−1^	

## 5. Conclusion

The proposed analytical method based on the use of Tb^3+^–simvastatin complex is simple and economic and can be successfully applied for sensitive and accurate determination of folic acid, progesterone, testosterone and vitamin D_3_ in different matrices including dosage forms, urine and serum. The analysis of the FCA, PGS, TST and Vit. D in biological samples can contribute to early diagnosis of some chronic diseases associated with their abnormal levels.

## Conflicts of interest

The authors declare that they have no known competing financial interests or personal relationships that could have appeared to influence the work reported in this paper.

## Supplementary Material
